# Broad-band Gaussian noise is most effective in improving motor performance and is most pleasant

**DOI:** 10.3389/fnhum.2014.00022

**Published:** 2014-02-03

**Authors:** Carlos Trenado, Areh Mikulić, Elias Manjarrez, Ignacio Mendez-Balbuena, Jürgen Schulte-Mönting, Frank Huethe, Marie-Claude Hepp-Reymond, Rumyana Kristeva

**Affiliations:** ^1^Department of Neurology and Neurophysiology, Albert-Ludwigs-UniversityFreiburg, Germany; ^2^Instituto de Fisiología, Benemérita Universidad Autonoma de PueblaPuebla, Mexico; ^3^Facultad de Psicología, Benemérita Universidad Autonoma de PueblaPuebla, Mexico; ^4^Institute for Medical Biometry and Medical Informatics, University of FreiburgFreiburg, Germany; ^5^Institute of Neuroinformatics, University of Zürich and ETH ZürichZurich, Switzerland

**Keywords:** noise, frequency, stochastic resonance, finger, motor, force, humans

## Abstract

Modern attempts to improve human performance focus on stochastic resonance (SR). SR is a phenomenon in non-linear systems characterized by a response increase of the system induced by a particular level of input noise. Recently, we reported that an optimum level of 0–15 Hz Gaussian noise applied to the human index finger improved static isometric force compensation. A possible explanation was a better sensorimotor integration caused by increase in sensitivity of peripheral receptors and/or of internal SR. The present study in 10 subjects compares SR effects in the performance of the same motor task and on pleasantness, by applying three Gaussian noises chosen on the sensitivity of the fingertip receptors (0–15 Hz mostly for Merkel receptors, 250–300 Hz for Pacini corpuscles and 0–300 Hz for all). We document that only the 0–300 Hz noise induced SR effect during the transitory phase of the task. In contrast, the motor performance was improved during the stationary phase for all three noise frequency bandwidths. This improvement was stronger for 0–300 Hz and 250–300 Hz than for 0–15 Hz noise. Further, we found higher degree of pleasantness for 0–300 Hz and 250–300 Hz noise bandwidths than for 0–15 Hz. Thus, we show that the most appropriate Gaussian noise that could be used in haptic gloves is the 0–300 Hz, as it improved motor performance during both stationary and transitory phases. In addition, this noise had the highest degree of pleasantness and thus reveals that the glabrous skin can also forward pleasant sensations.

## INTRODUCTION

Although often considered detrimental, noise can also have beneficial effects as demonstrated by the stochastic resonance (SR) phenomenon. The SR phenomenon occurs in non-linear systems in which an intermediate level of Gaussian noise enhances the response to weak signals (see recent review by Wiesenfeld and Moss, 1995; Gammaitoni et al., 1998; McDonnell and Abbott, 2009; McDonnell and Ward, 2011). Recent attempts to improve human performance have utilized this phenomenon. Most of these studies have applied tactile noise on feet or fingers, or electrical noise on the vestibular organs and also to the proprioceptive system (joint limbs such as the ankle and the knee) to improve balance and joint-position sense in healthy as well as motor impaired individuals (Gravelle et al., 2002; Priplata et al., 2002, 2006; Collins et al., 2003; Harry et al., 2005; Ross and Guskiewicz, 2006; Costa et al., 2007; Ross, 2007; Galica et al., 2009; Magalhaes and Kohn, 2011; Mulavara et al., 2011). We recently reported that an intermediate level of Gaussian noise (0–15 Hz) applied to the fingertip improved the motor performance during the stationary phase of a visuomotor task requiring isometric force compensation with the index finger (Mendez-Balbuena et al., 2012). In this report we had made the assumption that, among the fingertip cutaneous receptors involved in the task, the optimum level of noise did not activate Pacinian corpuscles with their lowest absolute threshold for frequencies of 250–300 Hz (Muniak et al., 2007; Johansson and Flanagan, 2009) but had activated Merkel disks (and to some extent Meissner receptors) responsive to low frequencies in the range of 5–15 Hz (Gardner et al., 2000). The question raised by our previous findings is whether higher frequencies of tactile noise (e.g., those frequencies preferentially activating Pacinian corpuscles) will be more effective in improving the motor performance than lower ones.

As Pacinian corpuscles and Merkel disks are not the only receptors activated by tactile noise, the present study investigates the effectiveness of optimum noise (ON) in three frequency bandwidths (0–15, 250–300, 0–300 Hz). Based on the theory of Collins et al. (1995) which suggests that SR effects are stronger when the number of neurons in a network of non-identical excitable units is increased, we predicted a best performance when a broad-band Gaussian noise activating all the skin (and proprioceptive) receptors is applied (0–300 Hz). To answer these questions an improved version of our previous manipulandum, able to generate noise up to 300 Hz, was developed and used. To test our predictions, we compared the behavioral performance (i.e., mean absolute deviation (MAD) and mean variation) during not only the stationary but also the transitory phases of the isometric compensation task under four experimental conditions: without noise (zero noise, ZN) and with superimposed optimal noise (ON) in three different bandwidths (0–15, 250–300, and 0–300 Hz).

In addition, we included a questionnaire on the subjective pleasantness of the applied SR noise, hypothesizing that the frequency bandwidth with the strongest effect on performance would be most pleasant.

## MATERIALS AND METHODS

### SUBJECTS

Ten subjects (mean age: 31.9 ± 13.7 years, nine female and one male) participated in the study. At the time of the experiment, all subjects were healthy and did not have any history of neurological disease. All subjects were right-handed as assessed by the Edinburgh Handedness Inventory (Oldfield, 1971). They gave written consent prior to the experiment in accordance with the declaration of Helsinki and all procedures were approved by the local ethics committee. All the subjects had participated in similar experiments before.

### EXPERIMENTAL PARADIGM

#### Paradigm

**FIGURE 1:**
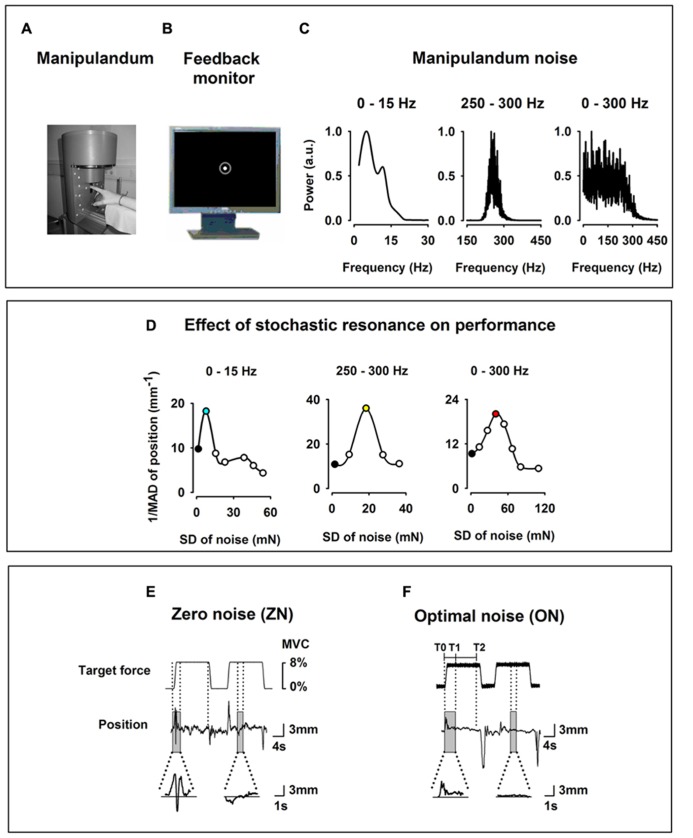
**Experimental setup.** **(A)** Home-made index finger manipulandum producing a target static force (SF; 8% of individual maximum voluntary contraction) on which noise in three frequency bandwidths (0–15, 250–300, and 0–300 Hz) is added. Profile of the target SF in **(E)** and in **(F)**. **(B)** Visual feedback of the finger position as a solid white dot within a green circle indicating the tolerance for position errors, displayed on a monitor in front of the subject. **(C)** Spectral power of the noise of the manipulandum in arbitrary units (au) for the three frequency bandwidths (0–15, 250–300, and 0–300 Hz). **(D)** Effect of the SR on the motor performance of one subject recorded prior to the experimental session for the three bandwidths and computed as the inverse of the mean absolute deviation of the finger position. Note the inverted U-shape like curve for all three bandwidths. During the experimental session only two noise levels were individually chosen, i.e., zero noise (ZN, black filled dots) and optimal noise (ON, filled dots for 0–15 Hz in turquoise for 0–15 Hz, in yellow for 250–300 Hz, and pink for 0–300 Hz). **(E,F)** Original curves for target force and finger position (representing the exerted force) for ZN **(E)** and ON **(F)** for the frequency bandwidth noise 0–300 Hz. Transitory phase of the task between markers T0 and T1 and stationary phase between markers T1 and T2. Note in the magnified position traces the better performance for ON than for ZN.

During the experimental session, the subject sat in an electrically shielded and dimly lit room. The right arm was supported by a splint and the subject was instructed to place the right hand over a sphere and his/her index finger in the ring of a home-made manipulandum (**Figure 1A**). The manipulandum was designed to produce a vertical force in upward direction on the ring. The subject had to compensate and maintain a target force (**Figure 1E**) quasi-isometrically by applying force at the level of the metacarpophalangeal joint in the opposite direction (downward).

#### Force profile

The target force shown in **Figure 1E** was set at 8% of the maximum voluntary contraction which was determined for each subject prior to the experiment. We used low force as it has been shown that motor cortical neurons are most sensitive to forces within a low force range (Hepp-Reymond et al., 1989). Each trial comprised three phases: a *1 s* *ramp phase* (rising cosine function to ensure a smooth start) followed by 12 s-period of *static force* (SF), followed by *downward ramp phase* (also cosine function) to ensure a smooth end of the generated force.

#### The manipulandum

The manipulandum used in this study is an improved version of the manipulandum used in Mendez-Balbuena et al. (2012). The construction of both manipulandi was based on a long travel subwoofer with cut diaphragm and a central spring. In the manipulandum used in Mendez-Balbuena et al. (2012) the central spring had been replaced with two linear bearings which were very light-weighted to save force. However, these bearings had some friction effects which were not negligible when using very low forces. In the improved manipulandum the friction force is reduced to almost zero by removing the linear bearings and using the central spring and a yarn transmitting pulling force to a lever. This lever in which the force sensor is assembled is pivot-mounted with a ball bearing with low friction. The force transducer used in this manipulandum is the force sensor KD45 (ME-Meßsysteme GmbH, Germany). The accuracy in combination with the sensor amplifier is better than 1% of full scale (5 N) and the linearity is 0.1%. The resolution of the position is better than 0.02 mm. The frequency bandwidth of the manipulandum is 0–470 Hz.

#### Visual feedback

The feedback of the force exerted by the subject was displayed as the position of a white dot (radius 2 mm) on a 19 inch monitor placed 100 cm in front of him/her (**Figure 1B**). This white dot moved within a fixed green circle (radius 6 mm including the line thickness of 2 mm) representing the range within which the white dot was allowed to move. When the force was applied to the ring and thus to the index finger, the subject had to compensate it by applying a quasi-isometric force in the opposite direction to maintain the white dot in the middle of the green circle. Thus, the feedback to the subject was a positional one, representing the exerted force. A finger displacement of 1 mm corresponded to 2.85 mm visual feedback. The tolerance for the positional errors was the green circle.

In each trial, segments in which the white point exited the green circle were excluded from further analysis. They represented approximately 1% of the total number of segments for each noise frequency bandwidth condition.

#### Experimental conditions

In the present experiment three bandwidths of Gaussian noise (0–15, 250–300, and 0–300 Hz) were applied to the manipulandum and added to the target force to test the SR effects on the performance. **Figure 1C** shows the spectral power of the noise for the three bandwidths. In the 0–15 Hz condition, 80% of the spectral power was contained in the frequency bandwidth between 0 and 15 Hz. In the 250–300 Hz condition as well in the 0–300 Hz, 80% of the noise energy was contained in these frequency ranges. The noise was generated by a MATLAB customized program which enabled various levels of noise intensity, beginning from ZN to high noise (200 mN).

Prior to the experiment three tests were performed:

*First*, the subjects had to make a few trials to get familiarized with the task and learn “what” to do and “how” to do it.

*Second*, to investigate the discriminative abilities of the subjects we defined the detection threshold (DT) for each of the three noise frequency bandwidths: 0–15, 0–300, and 250–300 Hz, using the following procedure. During a force trial with duration of 10 s at 8% of the maximum voluntary contraction (MVC), we increased the noise level in steps of 2 mN starting from zero, and defined the detection level when the subject reported that he/she felt the noise. For the determination of discrimination threshold, each administered noise level lasted 1 s. Thus 10 noise levels were given. The time interval between threshold determination trials was 3 min to prevent adaptation effects (Berglund and Berglund, 1970). This procedure was performed five times. The DT adopted for each subject corresponds to the average of the five trials performed.

*Third*, we defined for each subject the noise level which could be considered as ON for all three frequency bandwidths. In contrast to the strategy used in Mendez-Balbuena et al. (2012), where the ON selection was done subjectively by online observation of the position trace during several noise levels, we measured in the present experiment the ON level quantitatively. We made use of a MATLAB customized program that delivered a force at 8% MVC during 110 s and added noise levels in an incremental fashion (10 levels of noise were administered in steps of 10 mN, each one lasting 10 s, with an additional 10 s at the beginning comprising a base line period, a ramp phase to reach 8% MVC and a period to avoid transitory effects). Immediately after that we calculated the performance as a function of the noise level, i.e., the SR curve. **Figure 1D** shows examples of inverted U-like curve, which is the signature of the SR, as function of noise intensity for the three frequency bandwidths. We defined the ON as the noise level inducing the best performance, as measured by the smallest absolute deviation from 0. The procedure was repeated five times for each frequency bandwidth to ensure reliability of the measures. The ON adopted for each subject corresponded to the single level of noise that was most consistent within the five trials for each frequency bandwidth. The presentation of noise levels in an incremental fashion facilitated the determination of the ON as subjects could express what level of noise was subjectively more efficient in improving their performance, which in turn could also be corroborated with the information provided by the SR curve. The determination of ON was performed for the stationary period.

*During the experimental session*, two recording series of five trials each were collected for ZN and ON of the three frequency bandwidths, thus reaching 10 trials for each of them. ZN and the three ONs were presented in a pseudo-randomized fashion. To have the subjects sustaining attention during the experiment, the subjects had to report after each series of five trials whether the trials were without or with added noise. Furthermore, to avoid fatigue, rest intervals of 5–10 s were included between the trials. Rest periods of about 5 min between the series were given to avoid adaptation to the perception of the noise (Berglund and Berglund, 1970). The subjects were instructed to avoid any other movements and to fix their gaze on the visual feedback during the trials.

At the end of the experimental session the subject had to report the degree of pleasantness of the sensation elicited in the finger by the three noise bandwidths. The scale was: 0, unpleasant; 1, neutral; 2, pleasant.

### RECORDINGS

The force and displacement of the finger were recorded (bandpass DC – 500 Hz, sampling rate of 2000 Hz) and data were analyzed off-line.

## DATA ANALYSIS

### DETECTION THRESHOLD OF THE NOISE AND ON LEVEL

For DT and ON means, medians and quartiles were calculated and data presented as box plots. Scatter plots depicting the relationship between DT and ON for each subject and each frequency band were computed. A SEM bar graph for DT for each subject and each frequency bandwidth was also calculated.

### PLEASANTNESS OF THE NOISE

Means and SD were computed.

### MEAN DEVIATION (MD) AND MEAN ABSOLUTE DEVIATION

The data of the transitory and stationary phases of the task were analyzed separately. **Figure 1F** shows the markers for the transitory phase (T0 at the start of the ramp and T1 4 s after it) and for the stationary phase (from T1 to T2 at the end of the SF). The transitory phase lasted 4 s and the stationary phase 8 s. Data gained in the transitory phase from all trials were concatenated and the same was done for data of the stationary phase.

To test the effects of the SR phenomenon, we calculated the MD and MAD of the finger position magnitude.

The MD was computed on the basis of the formula

$$MD=\frac{1}{n}\overset{n}{\underset{i=1}{\Sigma }}x_{i}.$$

The MAD was computed on the basis of the formula:

$$MAD=\frac{1}{n}\overset{n}{\underset{i=1}{\Sigma }}\left|x_{i}\right|,$$

where *x_i_* is the value of finger position relative to the applied force at the sampling point *i*. MAD measures the deviation amplitude of the dot within the ring relative to the zero reference in both directions of the applied force.

### STATISTICAL ANALYSIS

#### Detection threshold, optimal noise, and pleasantness of the noise

To detect differences between the effects of the three bandwidths noise on DT, ON level, and pleasantness, the non-parametric Friedman test was applied. When the differences were significant a *post hoc* sign test was additionally performed.

#### Mean absolute deviation

The performance was measured as the inverse of the MAD of the finger position. ON referred to the noise level that yielded the smallest value of MAD (equivalently the largest values of its inverse 1/MAD) as defined quantitatively prior to the experiment (**Figure 1D**).

To prepare data for the statistical analysis, the individual MAD values whose distribution was large were first transformed logarithmically to yield symmetric distributions according to the formula:

$$\log_{10}\left(x\right).$$

The significance of the contrasts between ZN and ON was tested for the three frequency bandwidths and also the difference between them compared. In addition, our statistical model took both transitory and stationary phases into account. A two-way repeated measures ANOVA was computed on the transformed data with the factor *phase* (transitory and stationary) and *noise* (ZN, ON_0-15 Hz_, ON_250-300 Hz_, and ON_0-300 Hz_). Since we detected interactions between some factors we performed in addition the *post hoc* sign test for the transitory and stationary phases separately.

## RESULTS

### DETECTION THRESHOLDS FOR GAUSSIAN NOISE 0–15, 250–300, AND 0–300 Hz

**FIGURE 2:**
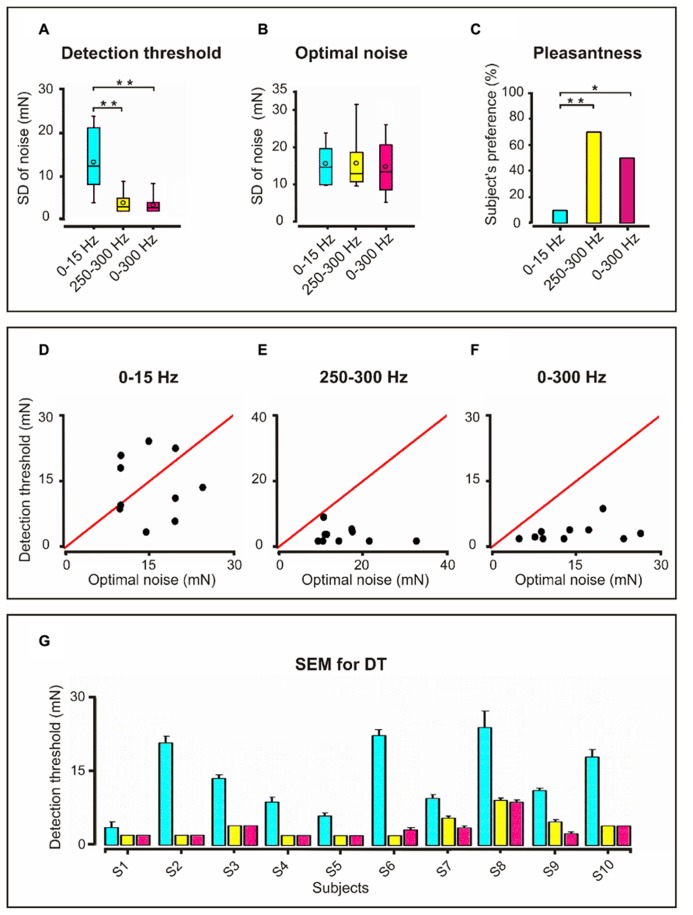
**Detection threshold, optimal noise and pleasantness for the three frequency bandwidths: Upper panel (A) Detection threshold in box plots with interquartile range displayed as the length of the box, with the median as a line inside the box and the minimal and maximal values as vertical lines.** Mean values shown as small unfilled circles within the boxplots. Higher detection threshold for 0–15 Hz compared to 250–300 Hz and 0–300 Hz bandwidths (**p = 0.002). **(B)** Boxplots for optimal noise showing similar noise levels for the three frequency bandwidths. **(C)** Higher degree of subjective pleasantness (percentage) for the 250–300 Hz and 0–300 Hz than for the 0–15 Hz noise bandwidth. *p = 0.02; **p = 0.003. Middle panel Graphs of detection threshold (DT) vs. Optimum noise (ON) for individual subjects corresponding to the three frequency bandwidths. The red line is the threshold. **(D)** 0–15 Hz; **(E)** 250–300 Hz and **(F)** 0–300 Hz. It is noticeable that for 250–300 and 0–300 Hz bandwidths all of the subjects exhibited supra-threshold SR (DTON) and six of them supra-threshold SR (DT < ON). *Lower panel*: **(G)** SEM bar graph depicting DT for each subject (S; 10 subjects) and for each frequency bandwidth. The color legend is the same as for the upper panel. Because the ON values are not averaged values there is no standard error for it. Note the higher standard error in DT for 0–15 Hz bandwidth.

In **Figure 2A** (upper panel) we display the results of the discriminative abilities of the ten subjects as measured by the DT for the three frequency bandwidths. The Friedman test revealed significant differences in detection level between the three frequency conditions (*p* = 0.0005, df = 2, *F* = 15.2). The *post hoc* sign test showed statistically significant differences between DT_0-15 Hz_ and both DT_250-300 Hz_ (*p* = 0.002) and DT_0-300 Hz_ (*p* = 0.002). No significant difference between DT_250-300 Hz_ and DT_0-300 Hz_ was observed. Thus, the results show higher sensitivity for noise bandwidths 250–300 Hz and 0–300 Hz.

### OPTIMUM NOISE LEVEL

**Figure 2B** (upper panel) shows the ON level for the three frequency bandwidths. No significant differences between the three ON noise levels were found.

### DEGREE OF PLEASANTNESS

**Figure 2C** (upper panel): the Friedman test revealed significant differences in the degree of pleasantness between the ON of the three frequency bandwidths (*p* = 0.004: df = 2, *F* = 11.15). As disclosed by the sign test the ON_0-300 Hz_ was more pleasant than the ON_0-15 Hz_ (*p* = 0.02) and the ON_250-300 Hz_ as well (*p* = 0.004). However, the degree of pleasantness for ON_0-300 Hz_ and ON_250-300 Hz_ did not differ. Interestingly, eight subjects reported that during ON_250-300 Hz_ and ON_0-300 Hz_ they felt the noise not only on the fingertip but that this feeling propagated proximally up to metacarpophalangeal joint and two subjects even up to the wrist.

### TYPE OF SR EXHIBITED BY THE SUBJECTS

Although no significant statistical relationship between DT and ON was found, it is interesting to note that while for the frequency bandwidths 250–300 and 0–300 Hz all subjects exhibited supra-threshold SR (**Figures 2E,F**; middle panel), for 0–15 Hz bandwidth four of the subjects exhibited sub-threshold SR and the other six supra-threshold SR (**Figure 2D**; middle panel). **Figure 2G** (lower panel) shows the standard error of the DT for each subject and frequency bandwidth.

### SR EFFECTS ON MOTOR PERFORMANCE

**FIGURE 3:**
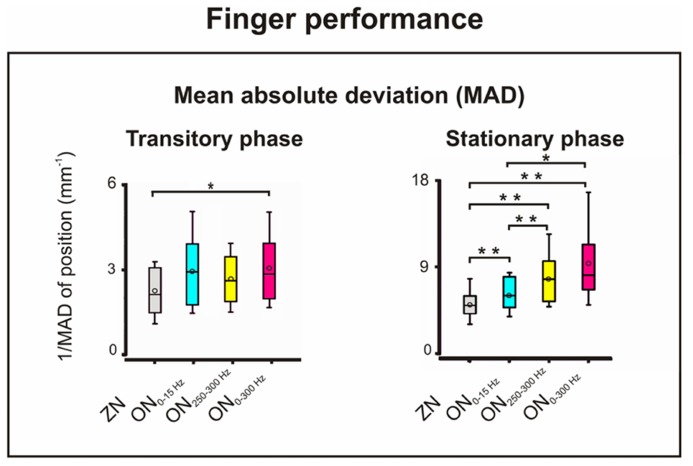
**Motor performance: mean absolute deviation (MAD) of the finger position for ZN and for ON for the three noise bandwidths during the transitory (left) and stationary (right) phases of the isometric compensation task.** During the transitory phase, stochastic resonance (SR) effect with better performance only for ON_0-300 Hz_ (*p = 0.01). During the stationary phase, SR effect for the three frequency bandwidths with best performance for ON_250-300 Hz_ and ON_0-300 Hz_. *p = 0.006; **p = 0.005.

To evaluate the effects of the three noise frequency bandwidths (ON_0-15 Hz_, ON_0-300 Hz_, and ON_250-300 Hz_) on the motor performance, MD, and MAD were computed and comparisons made between bandwidths and ZN for both transitory and stationary phases (**Figure 3**).

For the *MD*, the two-way repeated measures ANOVA (factors: phase and bandwidth of noise) showed significant effect for the factor *phase* only with larger MD in the transitory phase (*p* = 0.002, *F* = 19.8, df = 1). However, the interaction was not significant.

For the *MAD*, shown in **Figure 3**, the two-way ANOVA disclosed significant effects for the factors *phase* (*p* = 0.0001, *F* = 49.13, df = 1) and *noise bandwidth* (*p* = 0.0001, *F* = 15.05, df = 3), and for their interaction (*p* = 0.023, *F* = 3.74, df = 3). For the transitory phase, the sign-test only showed significant SR effect for ON_0-300 Hz_ vs. ZN (*p* = 0.012). For the stationary phase in contrast, it revealed SR improvement, i.e., decrease of MAD; for all three frequency bandwidths compared to ZN (*p* = 0.005). In addition, the ON_250-300 Hz_ induced greater improvement than the ON_0-15 Hz_ (*p* = 0.005). The same was true for the effect of ON_0-300 Hz_ which was stronger than that of ON_0-15 Hz_ (*p* = 0.007). No significant differences were observed between the SR effects of ON_250-300 Hz_ and ON_0-300 Hz_.

## DISCUSSION

In the present study we show for the first time that an individually measured optimal level of mechanical Gaussian noise in the frequency bandwidths of 0–300 Hz and in 250–300 Hz improves the performance in a visuomotor task requiring an isometric force compensation by the index finger. This improvement is greater than that observed with a bandwidth of 0–15 Hz and is most effective during the stationary phase of the task. The performance during the transitory phase was only improved by the 0–300 Hz noise. Further, we show that the 250–300 Hz and 0–300 Hz noises were perceived as more pleasant than the 0–15 Hz noise. Therefore, we propose that an individually determined ON in the broad bandwidth 0–300 Hz is the best candidate for the application to haptic gloves.

### WHY DO BANDWIDTHS OF 0–300 AND 250–300 Hz INDUCES BETTER PERFORMANCE THAN 0–15 Hz NOISE?

Studies comparing the SR effect induced by different noise bandwidths are scarce. In the vestibular system, not significant differences have been found in balance improvement when noise bandwidths of 1–2 and 0–30 Hz were compared (Mulavara et al., 2011).

Here we show that the 0–300 Hz ON induces a greater improvement in the sensorimotor task than the 0–15 Hz noise. This is consistent with a previous theoretical study demonstrating that SR effect increased when the number of neurons in a network was larger (Collins et al., 1995). As suggested in Mendez-Balbuena et al. (2012), the 0–15 Hz Gaussian noise most probably increased the sensitivity mainly of Merkel receptors and proprioceptors. One can expect that the 0–300 Hz noise increased in addition the sensitivity of the Pacini and of Meissner receptors. This in turn may produce an enhancement in SR with as a consequence an improvement in performance. But this cannot be the unique explanation since the SR effect with the 250–300 Hz noise, which selectively enhanced the sensitivity of the Pacini receptors, did not significantly differ from the SR effect produced by the 0–300 Hz noise. Therefore, we rather prefer the following complementary explanation. Manfredi et al. (2012) showed in humans that a vibration applied by a vibratory actuator to the fingertip traveled at least over the length of the finger and that the decay rate was dependent on the vibration frequency. Further, these authors reported that the resonant frequency of the skin matches the frequency at which the Pacinian receptors are most sensitive, i.e., 250–300 Hz and that such skin resonance lead to a twofold increase in response strength of the stimulated afferents.

In our study eight of the 10 subjects felt the ON of 250–300 and 0–300 Hz not only on the fingertip but up to the metacarpophalangeal joint and even up to the wrist. As our subjects reported such a proximal propagation only for the 0–300 and 250–300 Hz noises, i.e., for the frequency bandwidths including activation of the Pacinian receptors, it is most likely that a mechanism similar to the one described by Manfredi et al. (2012) plays a role. Most probably our Gaussian noise, including the frequencies at which the Pacinian receptors are most sensitive, leads to amplification of the neural response by skin resonance. Such skin resonance did not occur during noise 0–15 Hz, as the subjects did not feel any proximal propagation. The lack of significant difference in SR effects between 250–300 and 0–300 Hz noise strongly suggests that the contribution of the PC afferents is more important than that of the Merkel receptors whose sensitivity is increased by the 0–15 Hz noise. Aihara et al. (2010) have hypothesized that the optimization of the performance by externally applied noise is related to interaction between the internal noise of the system and the external noise. We thus suggest that, when the noise frequency bandwidth includes the frequencies at which the Pacinian receptors are most sensitive, the propagation of surface waves leads to an amplification of their response strength. This may be related to stronger interaction between the externally applied and the internal SR and therefore to a better sensorimotor integration and stronger SR effect.

Further, as we show that the DT for 250–300 Hz and 0–300 Hz noise was lower than for 0–15 Hz noise we suggest that the improvement in the isometric task is partly due to a local phenomenon of SR at the receptor level. It is thus quite plausible that the Pacinian receptors, activated by vibration frequencies of 250–300 Hz, are most contributing to the improvement. However, as stated in Leung et al. (2005) central factors must also play a role in psychophysical experiments. In EEG experiments we already have preliminary results showing higher cortical motor synchrony during ON as compared to ZN, as reflected in the higher EEG spectral power. Higher cortical spectral power reflects stronger sensorimotor integration (Mendez-Balbuena et al., 2013). The higher cortical motor synchrony drives more strongly the spinal motoneurons as reflected in the higher corticomuscular EEG–EMG coherence (Trenado et al., in preparation). In line with the involvement of central factors in the improvement of performance are some recent brain stimulation studies: Fertonani et al. (2011) using high frequency transcranial random noise stimulation (100–640 Hz) over the visual cortex improved behavioral performance in a visual task. Further, Feurra et al. (2011) using transcranial alternating current stimulation (tACS) over the primary somatosensory cortex in different frequency bandwidths showed that stimulation in alpha (10–14 Hz) and high gamma (52–70 Hz) produced a tactile sensation in the contralateral hand. It will be of great interest to investigate how Gaussian noise stimulation in different frequency bandwidths will selectively modulate cortical plasticity leading to an improvement in motor performance.

In our previous study (Mendez-Balbuena et al., 2012), the ON level for the 0–15 Hz frequency bandwidth was in a broader and higher range (SD of noise 0–200 mN) while in the present study the values are in the lower range (SD of noise 10–20 mN). This may be attributed to the less friction forces of the present manipulandum in comparison to the previous one. The friction forces act as an additional velocity-dependent noise stimulus. Such variability may also be explained by inter-individual differences.

### TYPE OF SR FOR NOISE BANDWIDTHS

In contrast to previous studies reporting on improvement of postural balance in humans through sub-threshold SR (Gravelle et al., 2002; Priplata et al., 2002, 2006; Collins et al., 2003), we found that for the frequency ranges 250–300 and 0–300 Hz all of the subjects exhibited supra-threshold SR, while for the range 0–15 Hz six subjects exhibited supra-threshold SR and four exhibited sub-threshold SR. This interesting observation is fully in line with other studies in the literature (Nozaki et al., 1999; Fallon and Morgan, 2005) showing the existence of both sub- and supra-threshold SR in the biological systems. Thus our findings have important translational aspect because they can be used in clinical studies for improving the performance in sensorimotor tasks.

### PLEASANTNESS OF THE 250–300 AND 0–300 Hz NOISE BANDWIDTHS

Löken et al. (2011) and McGlone et al. (2012) recently demonstrated that tactile stimulation of the hand glabrous skin can also elicit pleasant subjective experience. Based on the findings of Löken et al. (2011) that brush strokes at high speed were more effective to produce pleasure than low velocity brush strokes we expected that the 250–300 and 0–300 Hz noise bandwidths will induce the most pleasant sensation during the task.

The tactile sensation of pleasure is known to be mediated by a class of unmyelinated peripheral nerve fibers, termed CT afferents (C tactile) in the hairy skin (Zotterman, 1939; Nordin, 1990). However, the glabrous skin of the hand which does not contain CT afferents can also elicit pleasant subjective experience in response to tactile stimulation (Löken et al., 2011; McGlone et al., 2012). The tactile sensation of pleasure is mediated by fast-conducting myelinated A-beta afferents and is processed in the somatosensory cortex. In this context, our finding that pleasant tactile sensation was elicited by the application of mechanical noise on the glabrous skin of the finger can be attributed to the activation of myelinated A-beta afferents. We provide thus further evidence that sensory receptors from the glabrous finger skin also have the potential to elicit pleasant subjective experience for certain frequencies of tactile noise. Moreover, our results show that the most pleasant subjective experience occurs at higher frequencies of tactile noise, in agreement with the fact that brush strokes at a high speed on the glabrous skin of the hand were more effective to produce pleasure than brush strokes at low velocity (Löken et al., 2011). However, although higher frequencies of tactile noise were also more effective in improving the performance in our sensorimotor task, we suggest that the pleasantness produced by tactile noise did not contribute to induce this improvement. The mechanisms associated with a better performance may be related to SR at the level of the discriminative touch involving Pacinian receptors. It is tempting to suggest that among the myelinated A-beta afferents some of them are associated with pleasantness and others with discriminative touch. This suggestion is consistent with recent advances in the physiology of affective touch which has revealed that there is a pathway for pleasant touch from the glabrous skin in parallel to the pathway for discriminative touch (McGlone et al., 2007; Morrison et al., 2010; Klöcker et al., 2012). Nevertheless, further studies will be necessary to clarify whether just one pathway transmits both pleasant sensation and discriminative touch from the glabrous skin.

### IMPLICATIONS OF THE FINDINGS

The results from our analysis provide experimental evidence that the application of various noise frequencies differentially improves the performance in a force compensation task and also may induce the sensation of pleasantness. We expect that these new findings provide worthy information for the development of new haptic technology on the basis of discriminative and pleasant touch (Tsetserukou et al., 2009). However, one has to take into consideration that our manipulandum exerts slightly over-threshold vibration only in the vertical axis and that force is exerted in a single vertical direction. The findings of this special experimental condition are thus premature to be translated to a haptic glove as the use of haptic gloves implies multidirectional simultaneous forces. New investigation should apply SR phenomenon in tasks implying multidirectional forces to validate our suggestion.

As known from the literature the common practice in using vibratory SR is finding a threshold and using sub-threshold noise to enhance performance especially in balance control. A practical implication of our findings is that for 250–300 and 0–300 Hz frequency bandwidths the SR is supra-threshold and for 0–15 Hz bandwidth both sub- and supra-threshold. Thus the present study provides information what type of SR has to be used to increase performance in sensorimotor tasks.

## AUTHOR CONTRIBUTIONS

Rumyana Kristeva, Elias Manjarrez, Areh Mikulić, Jürgen Schulte-Mönting, Carlos Trenado, Ignacio Mendez-Balbuena, Frank Huethe, and Marie-Claude Hepp-Reymond designed the experiment and the methods. Carlos Trenado, Rumyana Kristeva, Ignacio Mendez-Balbuena, and Areh Mikulić carried out the experiment. Carlos Trenado, Jürgen Schulte-Mönting, and Rumyana Kristeva analyzed experimental data. Rumyana Kristeva, Carlos Trenado, Elias Manjarrez, Ignacio Mendez-Balbuena, Jürgen Schulte-Mönting, Frank Huethe, and Marie-Claude Hepp-Reymond interpreted data and wrote the manuscript. All authors approved the manuscript.

## Conflict of Interest Statement

The authors declare that the research was conducted in the absence of any commercial or financial relationships that could be construed as a potential conflict of interest.
